# Retinal Morphometric Markers of Crystallized and Fluid Intelligence Among Adults With Overweight and Obesity

**DOI:** 10.3389/fpsyg.2018.02650

**Published:** 2018-12-21

**Authors:** Alicia R. Jones, Connor M. Robbs, Caitlyn G. Edwards, Anne M. Walk, Sharon V. Thompson, Ginger E. Reeser, Hannah D. Holscher, Naiman A. Khan

**Affiliations:** ^1^Department of Kinesiology and Community Health, University of Illinois at Urbana-Champaign, Urbana, IL, United States; ^2^College of Liberal Arts and Sciences, University of Illinois at Urbana-Champaign, Urbana, IL, United States; ^3^Division of Nutritional Sciences, University of Illinois at Urbana-Champaign, Urbana, IL, United States; ^4^Department of Food Science and Human Nutrition, University of Illinois at Urbana-Champaign, Urbana, IL, United States; ^5^Neuroscience Program, University of Illinois at Urbana-Champaign, Urbana, IL, United States

**Keywords:** eye, optical coherence tomography, cognition, adiposity, retinal nerve fiber layer thickness, ganglion cell

## Abstract

**Objective:** To investigate the relationship between retinal morphometric measures and intellectual abilities among adults with overweight and obesity.

**Methods:** Adults between 25 and 45 years (*N* = 55, 38 females) with overweight or obesity (BMI ≥ 25.0 kg/m^2^) underwent an optical coherence tomography (OCT) scan to assess retinal nerve fiber layer (RNFL) volume, ganglion cell layer (GCL) volume, macular volume, and central foveal thickness. Dual-Energy X-ray absorptiometry was used to assess whole-body adiposity (% Fat). The Kaufman Brief Intelligence Test-2 was used to assess general intelligence (IQ), fluid, and crystallized intelligence. Hierarchical linear regression analyses were performed to examine relationships between adiposity and intelligence measures following adjustment of relevant demographic characteristics and degree of adiposity (i.e., % Fat).

**Results:** Although initial bivariate correlations indicated that % Fat was inversely related to fluid intelligence, this relationship was mitigated by inclusion of other demographic factors, including age, sex, and education level. Regression analyses for primary outcomes revealed that RNFL was positively related to IQ and fluid intelligence. However, only GCL was positively related to crystallized intelligence.

**Conclusion:** This work provides novel data linking specific retinal morphometric measures – assessed using OCT – to intellectual abilities among adults with overweight and obesity.

**Clinical Trial Registration:** www.clinicaltrials.gov, identifier NCT02740439.

## Introduction

Obesity prevalence is a growing global public health issue (Swinburn et al., 2011). In 2015, there were 604 million adults with obesity worldwide, representing a greater than twofold increase in prevalence since the 1980s (GBD Obesity Collaborators et al., 2017). In the United States, obesity is estimated to affect approximately 40% of the adult population (Hales et al., 2017). Excess fat mass or adiposity is known to directly contribute to a wide range of metabolic disorders and chronic diseases including type 2 diabetes and cardiovascular disease (Malnick and Knobler, 2006). However, overweight and obesity are also related to mood disorders including anxiety and depression and increasing evidence suggests that the detrimental consequences of obesity also extend to cognitive function and brain health (Romain et al., 2018) including greater risk for dementia in older age (Gustafson, 2006; Luchsinger and Gustafson, 2009).

While the underlying mechanisms remain unclear, evidence from magnetic resonance imaging (MRI) studies indicates that obesity is predictive of variations in brain structure and function that often accompany cognitive deficits including reduced synaptic plasticity (Erion et al., 2014), reduced processing speed (Sanz et al., 2013), and lower gray matter volume (Walther et al., 2009). Population-based studies have revealed that, akin to aging, increasing Body Mass Index (BMI) is longitudinally associated with declining gray matter volume (13 to 16% reduction per unit increase in BMI) in the temporal lobe (Gustafson D et al., 2004). Similarly, obesity has also been linked to MRI measures of white matter including hyperintensities (Gustafson D. R et al., 2004; Jagust et al., 2005; Stanek et al., 2011). Therefore, conventional neuroimaging techniques, primarily MRI, have revealed links between gray matter and white matter outcomes and obesity. However, the use of MRI presents many practical challenges including high financial costs, contraindications, susceptibility to movement artifacts, technical expertise necessary for scan acquisition and analyses, and limited mobility or accessibility for populations. Therefore, there is increasing need for determining the efficacy of alternative neuroimaging techniques with the requisite sensitivity to cognitive abilities and brain health, particularly among individuals with overweight or obesity.

Recent evidence indicates the morphometric measures of the human retina, studied using optical coherence tomography (OCT), have the potential to be utilized as markers of gray and white matter in the brain (Mutlu et al., 2017). Since the human retina is formed embryonically from neural tissue and is integrated into the neural system via the optic nerve, it is possible that structural abnormalities in brain tissue may be reflected in the retina (Chang et al., 2014; Mutlu et al., 2017). Additionally, imaging the retina, as proxy for brain, provides unique advantages since it can be visualized non-invasively at the cellular level due to its transparent nature, allowing for inexpensive testing of neurological biomarkers in clinical settings (Chang et al., 2014). OCT is a 3-dimensional retinal imaging technique that relies on low-coherence near infrared interferometry (Huang et al., 1991) to segment the various structural components of the retina including, but not limited to, the retinal nerve fiber layer (RNFL), ganglion cell layer (GCL), and macular volume and thickness. Although OCT is often used in clinical settings to detect abnormalities in the eye and monitor the progression of ocular diseases, retinal neurodegeneration has been recently correlated with cerebral atrophy suggesting that neuronal damage may occur simultaneously in the retina and throughout the brain (Ong et al., 2015). Additionally, the thickness of different layers of the retina are related to specific brain subcomponents of brain matter. For example, RNFL is composed of axons and RNFL thickness has been related to cerebral white matter. On the other hand, neuronal cell bodies comprise the GCL and may be reflective of cerebral gray matter (Mutlu et al., 2017). The RNFL relationship to white matter has received further support from studies among patients with Multiple Sclerosis demonstrating that RNFL is correlated with white matter tracts that are functionally separated from the visual system (Scheel et al., 2014). Several studies involving adults with Alzheimer’s have shown that these patients have reduced RNFL and GCL (Cheung et al., 2015; Thomson et al., 2015). Interestingly, thinner RNFL and GCL have also been associated with smaller temporal lobe structures including the hippocampus which is vital for memory and learning across the lifespan (Mutlu et al., 2017). Although emerging evidence points to the utility of OCT as a neuroimaging technique, only a limited number of studies have examined retinal morphometric measures and cognitive function. RNFL thickness has been positively associated with performance on the mini mental state examination among a large cohort of twins between 18 and 89 years (Jones-Odeh et al., 2016). Total macular volume and RNFL have also been associated with verbal intelligence and IQ among persons with MS (Ashtari et al., 2015). However, the extent to which different retinal layers correspond to aspects of intellectual abilities among individuals with overweight and obesity has not been directly examined.

Intelligence represents a critical cognitive ability known to support vital cognitive processes such as executive function and the acquisition of knowledge and learning (Colom et al., 2010). Intelligence can be conceptualized as general intelligence (i.e., intelligence quotient [IQ]) or its separable components of crystallized intelligence and fluid intelligence. Studying specific constructs of intelligence is important given that fluid and crystallized intelligence exhibit differential susceptibility to factors such as aging (Craik and Bialystok, 2006; Park and Reuter-Lorenz, 2009). Crystallized intelligence reflects the ability to use previously acquired knowledge and is therefore amenable to learning while fluid intelligence is thought to represent the ability to adapt to new situations (Cattell, 1963). In the context of obesity, studying these different measures of intelligence may provide insights into components of cognitive function that exhibit sensitivity to obesity-related cognitive impairments. However, to our knowledge, the relationship between retinal morphometric measures and intellectual ability among adults with overweight and obesity has not been previously studied.

Accordingly, the present work aimed to utilize OCT to assess the relationship between retinal morphometric measures and different constructs of intelligence among adults with overweight or obesity. Given prior evidence indicating that thicker RNFL and GCL are related to greater gray matter and white matter volumes among older adults, we hypothesized lower thickness in RNFL and GCL will be associated with poorer performance across all measures of intelligence (i.e., IQ, fluid, and crystallized).

## Materials and Methods

### Participants

Middle-aged adults (25–45 years) with overweight or obesity (BMI ≥ 25.0 kg/m^2^) were recruited from an ongoing dietary intervention (National Clinical Trail identifier NCT02740439). The data presented here were collected prior to the intervention phase of the study. Participants were recruited from the East-Central region of Illinois through e-mail listservs and flyers posted in public buildings. This study was carried out in accordance with the recommendations of the Declaration of Helsinki. The protocol was approved by the University of Illinois Institutional Review Board and written informed consent was obtained from all participants. Following informed consent, participants completed medical and demographic questionnaires. From these questionnaires, participants were excluded if they had a history of ocular (e.g., age-related macular degeneration), uncorrected vision, neurological disease, and/or chronic metabolic disease. Complete data was available for 53 participants. Participant characteristics are summarized in Table 1.

**Table 1:** Participant characteristics.

	All (*N* = 55)
Age, years	34.33 ± 0.82
Sex (F, M)	38,17
Body mass index, kg/m^2^	32.01 ± 0.78
25.0–29.9 kg/m^2^, n (%)	27 (49)
≥30.0, n (%)	28 (51)
Whole body adiposity, %	39.08 ± 1.21
Intelligence quotient	106.13 ± 1.77
Crystallized intelligence	102.09 ± 2.10
Fluid intelligence	108.62 ± 1.63
Education level	
High school, n (%)	2 (4)
Undergraduate College Degree, n (%)	19 (34)
Advanced College Degree, n (%)	34 (62)
^a^Macular volume (mm^3^)	3.11 ± 0.15
^a^RNFL volume (mm^3^)	0.21 ± 0.02
^a^GCL volume (mm^3^)	0.45 ± 0.04
^a^Center foveal thickness (μm)	222.51 ± 16.73


*Data presented as mean ± SD wherever unless indicated otherwise. ^a^Based of average of both left and right eyes available for 53 subjects. RNFL, retinal nerve fiber layer; GCL, ganglion cell layer.*

### Overall Procedure

Data were collected over two visits to the laboratory. During the first session, following written informed consent and screening, trained researchers administered the Kaufman Brief Intelligence Test Second Edition (KBIT-2) (Naugle et al., 1993; Wang and Kaufman, 1993) and participants underwent OCT assessment in both eyes. During the second session, participants arrived following a 10-h fast and underwent a whole body Dual-Energy X-ray Absorptiometry (DXA) scan.

### Retinal Morphometry Assessment

**FIGURE 1:**
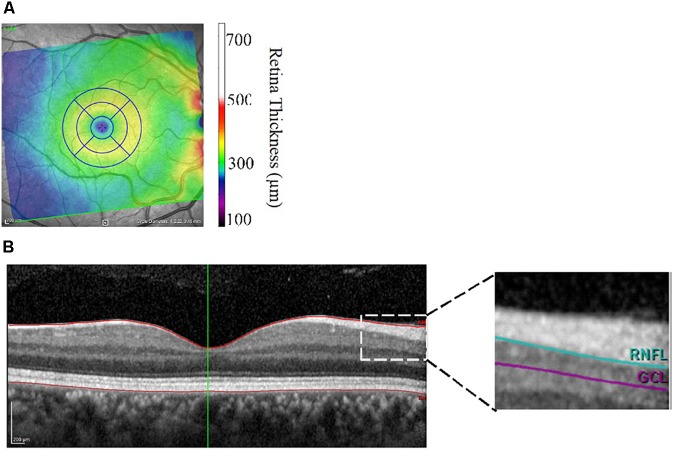
**(A,B)** Image output from Heidelberg Spectralis Optical Coherence Tomography focusing on the macula **(A)** and cross-sectional view of the retinal layers **(B)**. Central foveal thickness was assessed at the foveal pit. RNFL, retinal nerve fiber layer; GCL, ganglion cell layer thickness.

Retinal morphometric data were assessed using retinal images collected with the Heidelberg Engineering Spectralis Optical Coherence Tomography (SD-OCT; Heidelberg Engineering, Heidelberg, Germany). The principles of the SD-OCT technique have been previously discussed (Galetta et al., 2011). Briefly, the SD-OCT procedure relies on the interferometer to transmit low-coherence infrared light through the pupil and the layers of the retina. This SD-OCT device utilizes a class one laser to emit infrared light at 870 nm through a super luminescence diode. Results were obtained using the central, inner, and outer rings centered around the fovea with respective diameters of 1, 2.22, and 3.45 mm. Macular, RNFL, and GCL volume were all obtained using the 3.45 diameter circle. Center Foveal Thickness was found by taking the thickness measurement at the center-most point at the foveal pit. Volume and thickness were assessed using Heidelberg software (version: 6.0.11.0). Each scan was manually segmented to account for blood vessels by trained researchers. Figures 1A,B illustrate the retinal layers examined. Data were collected on both eyes and there was a high degree of correlation between the thickness and volume measures between right and left eyes (*r*’s between 0.71 and 0.97 all *P*’s < 0.01). Therefore, average values of the left and right eyes were used in the statistical analyses.

### Intelligence Assessment

Kaufman Brief Intelligence Test Second Edition has been nationally normed for ages 4–90 years to assess general intellectual abilities (IQ) (Wang and Kaufman, 1993). The test is divided into three sub-tests and takes approximately 25–30 min for adults to complete. The crystallized intelligence measure includes the Verbal Knowledge and Riddle subtests. The fluid intelligence measure is assessed with the Matrices subtest. The Verbal Knowledge test includes 60 questions where the participant responds by choosing which image is most associated with the word or question spoken by the researcher. The Riddle subtest consists of 48 riddles with single word answers. The matrices test has 46 multiple choice problems where the participant must choose which of 6 pictures are most associated with the single stimulus picture or best completes a 2×2, 2×3, or 3×3 matrix. In each of the subtests, correct answers are given a score of 1 and the total scores are converted into standard scores with a maximum of 100.

### Anthropometrics and Adiposity Assessment

Adipose tissue was measured by using a Hologic Horizon W bone densitometer (APEX Software version 5.6.0.5; Hologic, Bedford, MA, United States). Whole body percent body fat (% Fat), was assessed using Hologic software, as previously described (Baumgartner et al., 2017).

### Statistical Analysis

Pearson correlation analyses were conducted to determine the contribution of demographic and retinal morphometric measures to the intelligence outcomes. Stepwise hierarchical linear regression models were used to examine the contribution of retinal morphology measures to intelligence measures following adjustment for potential confounding variables. Age, sex, education, and % Fat were entered as step 1 control variables and morphometric measures were added at step 2 in the analyses. The significance of the change in the *R^2^-*value between the two steps was used to evaluate the improvement in the variance explained once retinal measures were included. The independent contribution of each retinal morphometric measure was assessed by studying the β weight and significance at step 2 when explaining variance in intelligence outcomes beyond that of the demographic variables and adiposity. Data were analyzed using SPSS (SPSS v. 24, Chicago, IL, United States) with an *alpha* threshold of *p* = 0.05.

## Results

The sample consisted of 55 participants, ages 25–45 (*M* = 34.33 ± 0.82 years) and was predominantly comprised of females (*n* = 38). Approximately half of the sample was comprised of individuals with a BMI between 25 and 29.9 kg/m^2^ (49%) and the other half (51%) of the sample had a BMI ≥ 30 kg/m^2^. Majority of the study participants had obtained higher education or advanced college degrees (62%).

### Bivariate Correlations

Preliminary Pearson bivariate correlations are summarized in Table 2. Sex (males coded as 1, females coded as 0) was negatively correlated with % Fat (*r* = -0.76, *p* ≤ 0.01), and positively correlated with macular volume (*r* = 0.34, *p* = 0.01), RNFL volume (*r* = 0.35, *p* = 0.01), IQ (*r* = 0.34, *p* = 0.01), and fluid intelligence (*r* = 0.39, *p* ≤ 0.01). Age was positively correlated with center foveal thickness (*r* = 0.29, *p* = 0.03). % Fat was negatively correlated with fluid intelligence (*r* = -0.31, *p* = 0.02) and macular volume (*r* = -0.27, *p* = 0.05) but trended for IQ (*r* = -0.25, *p* = 0.07). Education level was not significantly related to any intelligence or demographic variable (all *p’*s > 0.09).

**Table 2:** Bivariate correlations between participant characteristics, retinal morphology, and intellectual ability.

			1	2	3	4	5	6	7	8	9	10	11
1.	Age	*r*											
		*p*											
2.	Sex	*r*	0.06										
		*p*	0.69										
3.	Education	*r*	-0.13	0.01									
		*p*	0.34	0.96									
4.	% Fat	*r*	-0.04	-0.76**	-0.06								
		*p*	0.79	<0.01	0.68								
5.	Macular volume	*r*	0.14	0.34*	0.03	-0.27*							
		*p*	0.32	0.01	0.81	0.05							
6.	RNFL volume	*r*	0.19	0.35*	0.24^†^	-0.16	0.49**						
		*p*	0.17	0.01	0.09	0.24	<0.01						
7.	GCL volume	*r*	0.16	0.21	0.03	0.01	0.73**	0.56**					
		*p*	0.24	0.13	0.85	0.95	<0.01	<0.01					
8.	Center foveal thickness	*r*	0.29*	0.25^†^	-0.14	-0.14	0.39**	0.24	0.28*				
		*p*	0.03	0.07	0.33	0.32	<0.01	0.09	0.04				
9.	Intelligence quotient	*r*	-0.05	0.34*	0.10	-0.25^†^	0.23^†^	0.462**	0.32*	0.27*			
		*p*	0.74	0.01	0.45	0.07	0.09	<0.01	0.02	0.05			
10.	Crystallized intelligence	*r*	-0.01	0.23	0.08	-0.15	0.31*	0.40**	0.43**	0.29*	0.89**		
		*p*	0.97	0.10	0.57	0.27	0.02	<0.01	<0.01	0.04	<0.01		
11.	Fluid intelligence	*r*	-0.03	0.39**	0.11	-0.31*	0.08	0.42**	0.10	0.18	0.81**	0.45**	
		*p*	0.84	<0.01	0.43	0.02	0.59	<0.01	0.49	0.19	<0.01	<0.01	


*^†^p < 0.10; ^∗^p < 0.05; ^∗∗^p < 0.01. RNFL, retinal nerve fiber layer; GCL, ganglion cell layer.*

### Regression Analyses

A summary of the regression analyses for each measure of intelligence is provided in Table 3. Step 1 in each of the models adjusted for age, sex, education level, and % Fat and was not statistically significant for IQ (Δ*R*^2^ = 0.15, *p* = 0.10) and crystallized intelligence (Δ*R*^2^ = 0.07, *p* = 0.47). However, step 1 was a significant predictor for fluid intelligence (Δ*R*^2^ = 0.18, *p* = 0.05). Additionally, among model 1 variables, sex was a significant predictor of IQ (β = 0.43, *p* = 0.05) and fluid intelligence (β = 0.43, *p* = 0.05); however, this relationship was mitigated in step 2 after retinal measures were included for IQ (β = 0.10, *p* = 0.66) and fluid intelligence (β = 0.22, *p* = 0.32).

**Table 3:** Summary of hierarchical regression analysis for intelligence measures.

	Intelligence quotient				Crystallized intelligence				Fluid intelligence			
	β	*p*	Δ*R*^2^	Model *p*	β	*p*	Δ*R*^2^	Model *p*	β	*p*	Δ*R*^2^	Model *p*
**Step 1**												
Age	-0.08	0.56	0.15	0.10	-0.04	0.78	0.07	0.47	-0.05	0.70	0.18	0.05
Sex	0.43*	0.05			0.33	0.15			0.43*	0.05		
Education	0.10	0.48			0.09	0.54			0.09	0.52		
% Fat	0.09	0.67			0.11	0.62			-0.03	0.91		
**Step 2**			0.20	0.01			0.23	0.04			0.16	0.02
TMV	-0.29	0.16			-0.21	0.32			-0.30	0.16		
RNFL	0.37*	0.03			0.21	0.22			0.46**	<0.01		
GCL	0.27	0.19			0.44*	0.05			-0.11	0.96		
CFT	0.24^†^	0.10			0.24^†^	0.11			0.16	0.27		


*^†^p < 0.10; ^∗^p < 0.05; ^∗∗^p < 0.01. TMV, macular volume; RNFL, retinal nerve fiber layer; GCL, ganglion cell layer; CFT, central foveal thickness; % Fat; whole body adiposity.*

All models were significant once retinal measures were included at step 2 (*p*’s 0.01 to 0.04). RNFL volume was positively related to IQ (β = 0.37, *p* = 0.03), whereas central foveal thickness (β = 0.24, *p* = 0.10) only approached statistical significance. Additionally, GCL was the only independent contributor to the variability in crystallized intelligence (β = 0.44, *p* = 0.05) whereas central foveal thickness approached statistical significance (β = 0.24, *p* = 0.10) in relation to IQ. Finally, RNFL volume was the only independent contributor to variance in fluid intelligence (β = 0.46, *p* < 0.01).

## Discussion

This study aimed to determine the relationship between retinal morphometric measures and intellectual abilities among adults with overweight and obesity. Consistent with our *a priori* hypothesis, we observed that RNFL and GCL volume were significantly related to higher intellectual ability. Interestingly, these relationships were selective in that RNFL and GCL were related to fluid and crystallized intelligence, respectively. On the other hand, we observed that greater macular volume and CFT were not significant predictors of any of our measures of intellectual abilities. However, the influence of CFT on IQ and crystallized intelligence approached statistical significance. Overall, these data indicate that OCT-derived retinal measures are related to intellectual abilities among adults with overweight and obesity.

Obesity has been shown to be related to lower gray matter across several brain regions, including prefrontal cortex, temporal, occipital cortex, amygdala, and cerebellum, even after adjusting for obesity-related comorbidities (Kharabian Masouleh et al., 2016). Given that the retina shares developmental, physiological, and anatomical features with the brain, retinal imaging has emerged as an alternative approach to imaging the neural structures (de la Monte et al., 2009; Kamran Ikram et al., 2012; Ong et al., 2015). The efficacy for using OCT for neural imaging has gained particular empirical support from studies in neurodegenerative diseases. For example, histopathological and clinical studies have shown that patients with Alzheimer’s disease have reduced GCL and RNFL thickness compared to controls (Coppola et al., 2015). Recent work has related RNFL and GCL thinning to global and regional cerebral atrophy using MRI among neurologically healthy adults (Ong et al., 2015; Casaletto et al., 2017). Ong et al. (2015) studied a sample of 60–80-year-olds (*N* = 164) and observed that GCL thinning was selectively related to reduction in occipital and temporal lobe gray matter volume, while no relationships were observed with white matter. Additionally, in a large population based study (*N* = 2,124), Mutlu et al. (2017) observed that thinner RNFL and GCL were associated with poorer white-matter microstructure. The RNFL and GCL are known to be components of the ganglion cell complex with the RNFL comprising axons and the GCL signifying cell bodies. Therefore, it is possible the RNFL may correspond to cerebral white matter while the GCL may reflect cerebral gray matter integrity (Mutlu et al., 2017). However, to our knowledge, this is the first study to implicate thinner retinal morphometric measures in poorer intellectual ability among adults with overweight and obesity.

Although a considerable body of literature has examined the influence of obesity on brain structure and cognitive function (Pannacciulli et al., 2006; Smith et al., 2011), the influence of obesity on measures of intelligence has received comparatively less attention. Studying neuroimaging markers of intellectual abilities is important because intelligence supports higher-order mental processes such as executive function (also known as cognitive control) as well as the acquisition of knowledge and learning across the lifespan (Colom et al., 2010). Data from the present work indicated increasing adiposity was inversely related to fluid intelligence. However, this relationship was no longer significant once other demographic factors were included in step 1 of the regression models. Fluid intelligence represents abstract reasoning and problem solving abilities and is an important predictor for lifetime trajectories of cognition and physical and mental health (Gottfredson, 1997). Importantly, the retinal morphometric measure of RNFL was the primary predictive variable for fluid intelligence. Previous neuroimaging work has shown that abnormalities in white matter influence a variety of cognitive functions, particularly under demyelinating diseases such as multiple sclerosis (Kail, 1998). White matter integrity, as indicated by fractional anisotropy, has also been shown to be related to general intellectual abilities and fluid intelligence (Yu et al., 2008; Haász et al., 2013). If the RNFL is reflective of white matter integrity, the findings of the current study are consistent with these aforementioned studies since we also observed a significant positive relationship between RNFL and fluid intelligence. On the other hand we observed that GCL volume was selectively related to crystallized intelligence. Crystalized intelligence is distinct from fluid intelligence because it refers to the ability to retrieve and use information that has been acquired throughout life (Horn and Cattell, 1968). Unlike fluid intelligence, crystallized intelligence does not exhibit susceptibility to aging (Park and Reuter-Lorenz, 2009). The implication of the finding from the current study is that GCL reflects intellectual abilities that are acquired through learning across the lifespan. Future studies are needed to determine whether changes in obesity and fat distribution differentially compromise particular intellectual abilities during development and aging.

While GCL and RNFL were found to be predictive of intellectual abilities, we did not observe significant correlations between measures of macular volume and central foveal thickness and intelligence. It is worth noting that the association among foveal thickness, IQ, and crystallized intelligence approached statistical significance. It is possible that the influence of foveal thickness on intellectual ability is comparatively smaller, relative to GCL and RNFL, and our sample was not adequately powered to detect the relationships. Nevertheless, the patterns observed (i.e., potential relationships among foveal thickness, IQ, and crystallized intelligence) would be similar to those observed for GCL. Thus, greater foveal thickness may be protective of intellectual abilities thought to be acquired by learning through the lifespan. Given that previous work has shown that foveal thickness is associated with macular pigment optical density or the accumulation of macular carotenoids (Liew et al., 2006), future intervention trials are necessary to determine the susceptibility of the fovea to dietary intake and its implications for intellectual abilities.

Although the present study provides novel data linking intellectual abilities to retinal morphometric measures assessed by OCT, there are several limitations worth considering. Longitudinal research studies are necessary to characterize changes in retinal measures and intellectual abilities over extended periods of time. Additionally, our study lacked a comparator group of individuals with a healthy weight status. Improving the heterogeneity of the sample by including individuals with varying weight status would provide more comprehensive insights into the relationship between obesity, intellectual abilities, and retinal measures. Finally, we did not account for genetic factors that may contribute to retinal morphometric measures. For example, Jones-Odeh et al. (2016) examined a large cohort of twins in the United Kingdom and learned that RNFL thickness was highly heritable (82%). Markers of vascular health in the retina have also been previously linked to neuropsychological functioning at midlife should be accounted for in future research (Shalev et al., 2013). Additionally, other lifestyle factors (e.g., diet and physical activity) have the potential to contribute to intellectual abilities and/or retinal morphology and warrant examination in future studies.

In conclusion, these findings provide cross-sectional evidence supporting the efficacy or utility of retinal morphometric measures – as measured by OCT – to study intellectual abilities among adults with overweight and obesity. Importantly, we were able to demonstrate these relationships in a sample of middle-aged adults since previous work has predominantly focused on older adults and individuals with dementia. Selective relationships were observed between particular retinal measures and different intellectual abilities, known to be differentially affected by aging. These data may set the stage to develop future research into the interaction between aging and weight status and their influence on gray and white matter and different constructs of intelligence.

## Author Contributions

AJ, CR, CE, ST, and GR collected the data and contributed to the manuscript draft. AW interpreted the results and contributed to the manuscript development. HH and NK conceptualized the study, interpreted the results, and contributed to the manuscript draft.

## Conflict of Interest Statement

The authors declare that the research was conducted in the absence of any commercial or financial relationships that could be construed as a potential conflict of interest.
